# Multi-Organ NMR Metabolomics to Assess In Vivo Overall Metabolic Impact of Cisplatin in Mice

**DOI:** 10.3390/metabo9110279

**Published:** 2019-11-13

**Authors:** Tatiana J. Carneiro, Rita Araújo, Martin Vojtek, Salomé Gonçalves-Monteiro, Carmen Diniz, Ana L.M. Batista de Carvalho, Maria Paula M. Marques, Ana M. Gil

**Affiliations:** 1 Department of Chemistry and CICECO–Aveiro Institute of Materials, University of Aveiro, 3810-193 Aveiro, Portugal; tatiana.joao@ua.pt (T.J.C.); anarita.asilva@ua.pt (R.A.); 2 LAQV/REQUIMTE, Department of Drug Sciences, Laboratory of Pharmacology, Faculty of Pharmacy, University of Porto, 4150-755 Porto, Portugal; matovoj@gmail.com (M.V.); salomegmonteiro@hotmail.com (S.G.-M.); cdiniz@ff.up.pt (C.D.); 3 “Química-Física Molecular”, University of Coimbra, 3004-535 Coimbra, Portugalpmc@ci.uc.pt (M.P.M.M.); 4 Department of Life Sciences, Faculty of Science and Technology, University of Coimbra, 3000-456 Coimbra, Portugal

**Keywords:** cisplatin, mice, mouse model, in vivo, NMR, metabolomics, metabonomics, kidney, liver, breast tissue

## Abstract

This work describes, to our knowledge, the first NMR metabolomics analysis of mice kidney, liver, and breast tissue in response to cisplatin exposure, in search of early metabolic signatures of cisplatin biotoxicity. Balb/c mice were exposed to a single 3.5 mg/kg dose of cisplatin and then euthanized; organs (kidney, liver, breast tissue) were collected at 1, 12, and 48 h. Polar tissue extracts were analyzed by NMR spectroscopy, and the resulting spectra were studied by multivariate and univariate analyses. The results enabled the identification of the most significant deviant metabolite levels at each time point, and for each tissue type, and showed that the largest metabolic impact occurs for kidney, as early as 1 h post-injection. Kidney tissue showed a marked depletion in several amino acids, comprised in an overall 13-metabolites signature. The highest number of changes in all tissues was noted at 12 h, although many of those recovered to control levels at 48 h, with the exception of some persistently deviant tissue-specific metabolites, thus enabling the identification of relatively longer-term effects of cDDP. This work reports, for the first time, early (1–48 h) concomitant effects of cDDP in kidney, liver, and breast tissue metabolism, thus contributing to the understanding of multi-organ cDDP biotoxicity.

## 1. Introduction

Cisplatin (cDDP) is a platinum(II)-containing drug (cis-[Pt(NH_3_)_2_Cl_2_]) which is well known for its anticancer properties [1,2]. The impact of cDDP in cell division was observed for the first time in 1965 (in *E. coli*) and the compound was approved as an antineoplastic agent in 1978 [2]. CDDP is presently used, often in combination with other drugs, for treatment of many different types of cancer, e.g., head and neck, lung, testicular, ovarian, and bladder cancer [1,3]. However, its clinical use is limited to some extent due to frequently acquired drug resistance and severe cytotoxic effects such as nephrotoxicity, hepatotoxicity, cardiotoxicity, neurotoxicity, and bone marrow toxicity [3]. Attempts to overcome these drawbacks have been made through the development of several other Pt (or other metal)-based drugs, such as carboplatin (a Pt complex with bidentate cyclobutane dicarboxylic acid replacing the chloride ions), which represents one of the most successful [1]. Furthermore, the combination of cDDP with other chemotherapeutic agents, such as taxanes (paclitaxel), anthracyclines (doxorubicin), or antimetabolites (tegafur-uracil, gemcitabine, capecitabine), has been extensively used to reduce cDDP deleterious side effects and acquired resistance, while increasing patient compliance to treatment [1,4,5].

CDDP biotoxicity is known to lead to considerable deviant effects at the intracellular level (namely DNA damage, organelle dysfunction, oxidative stress, or inflammation), as well as on the plasma membrane, which then translates into deviations in endogenous metabolic status [1,2]. This abnormal metabolic behavior may potentially unveil new early biomarkers of cDDP biotoxicity, which may enable timely recognition and management in a clinical context; metabolomics is an enticing means to identify such biomarkers. Indeed, metabolomics has emerged as a promising approach to evaluate in vitro and in vivo responses to drugs [6,7]. This strategy provides valuable insight into the metabolic perturbations produced locally (in affected tissues) and at the systemic level, as viewed through the metabolome of biofluids [8,9]. In the context of response to cDDP, most studies have addressed its role in cancer treatment, with fewer reports dedicated to cDDP toxicity alone (i.e., in healthy cell lines or animal models). In cancer treatment with cDDP, metabolomics has been applied to several human cancer cell lines in search of biomarkers of chemotherapy efficacy (lung [10,11], breast [12,13], bone [14,15], brain [16], and ovarian [17,18]) or resistance (ovarian [17,18,19], ovarian and lung [20], breast [21]). CDDP treatment efficacy and nephrotoxicity have also been assessed through kidney tissue metabolomics in a mouse model of metastatic melanoma [22], and biofluids, namely serum/plasma [23,24,25] and urine [26], in a model of lung cancer, both for cDDP sole [24,25] and combined administration [23,24].

Regarding the use of metabolomics to improve the understanding of cDDP biotoxicity mechanisms, some studies have addressed healthy liver [27], lung [10], and kidney [28] cell lines. In particular, human liver L02 cells incubated with cDDP and analyzed (upon extraction) by NMR metabolomics [27] showed drug effects on cell metabolism for variable orders of magnitude of drug concentrations (1 nM to 1 mM), noting that the highest dose induced pronounced perturbations in the levels of amino acids and nucleotides. NMR metabolomics was also used to analyze the (polar) metabolic profiles of human healthy lung cells (MRC5 cell line), compared to cancer cells (A549 cell line), upon treatment with several chemotherapeutics agents, including cDDP [10]. The heathy cell line showed decreasing trends in several metabolites (e.g., branched-chain amino acids (BCAAs), glutamine and glycine, lactate, glutathione, and taurine) as an indication of drug toxicity effects, possibly underlying the response of cancer cells to cDDP treatment. In the case of renal cell lines, a study of the human renal proximal tubular RPTEC/TERT1 cell line was carried out through ‘multi-omics’ approaches, namely transcriptomics, proteomics, and metabolomics [28]. Both cellular supernatants and lysates analyses revealed distinct perturbations, particularly in relation to choline, fatty acids, and glutathione metabolism. Furthermore, metabolomics has also focused on healthy rodent models to address tissue damage upon cDDP exposure [29] and to help assess nephrotoxicity, since this is the major dose-limiting factor related to cDDP treatment [5]. Nephrotoxicity-related metabolic changes have been observed in the biofluids (blood plasma/serum and urine) of rodent models, reflecting clear dose- and time-dependent deviant metabolic responses to cisplatin exposure [30,31,32,33,34,35,36,37,38,39,40,41]. Although biofluids metabolic profiles carry valuable information on the systemic metabolic response to cDDP, and may be an important source of non-invasive early markers of drug toxicity, tissue metabolic analyses (including but not restricted to kidney) may provide important information on local deviant metabolism and interorgan metabolic interplays. To our knowledge, only cisplatin-exposed kidney tissue has been studied through metabolomics, notably using MS-based untargeted metabolomics [3,42,43]. The first studies addressed Sprague-Dawley (SD) rats, with an early report monitoring both urine and kidney tissue after 1, 5, and 28 days post cDDP injection (0.5 mg/kg) [42]. An interesting result related to the early observation of altered levels of polyamines and amino acids (namely BCCAs) in urine, whereas tissue revealed depleted levels of several amino acids (consistently with their increased excretion) and nucleosides. The metabolic heterogeneity in kidney was addressed in two further metabolomic studies, either considering medulla and cortex measurements separately (using 2.5, 5, and 10 mg/kg cDDP) [43], or by using MS imaging (MSI) techniques in tandem with LC-MS (using 30 mg/kg cDDP) [3]. Upon 7 days post-injection with different cisplatin doses [43], metabolic changes were shown to be more significant and dose-dependent in kidney medulla, compared to cortex. Many amino acids were found to be down-regulated in both kidney sections, in tandem with up-regulation of fatty acids (FA), diacylglycerols (DG), ceramide, and sphingosine, along with a set of metabolites noted to distinguish the metabolic profiles of medulla and cortex. On a shorter time-scale (3–72 h), in a study of mice exposed to a large cDDP dose (30 mg/kg b.w.) [3], significant region-specific metabolic differences were confirmed, and early changes (at times < 24 h) were noted in several compounds, such as amino acids (up-regulated, in apparent contradiction with previous observations), nucleotides, and intermediates of folate metabolism.

In this work, a ^1^H NMR-based metabolomics strategy was carried out, for the first time to our knowledge, to assess the impact of cisplatin on the metabolism of the kidney, adding the novel study of the concomitant metabolic changes in the liver and breast tissues of the same healthy mice. The latter type of tissue should be particularly useful as a control matrix for studies of breast cancer animal models treated with cisplatin or other drugs. As a technique which is complementary to MS-based methods, NMR metabolomics should confirm previous observations in kidney tissue (for comparable timescales and drug doses), while unveiling novel metabolic changes, not only in kidney, but also in liver and breast tissue. Focusing on a very short timescale of exposure to cDDP (1 to 48 h), this study provides an early dynamic metabolic picture of the interplay of different organs in responding to the drug, thus unveiling potential early multi-organ metabolic markers of cDDP biotoxicity.

## 2. Results

### 2.1. Typical ^1^H NMR Spectra of Aqueous Extracts of Mice Kidney, Liver and Breast Tissue

**Figure 1:**
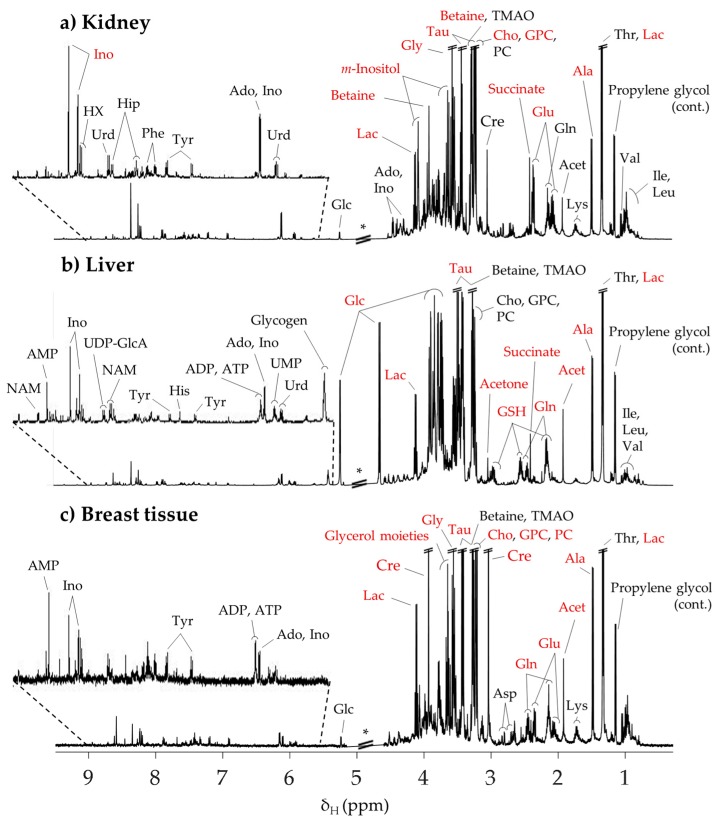
Typical 500 MHz ^1^H NMR spectra of aqueous extracts of (**a**) kidney, (**b**) liver, and (**c**) breast tissue from the control group (BALB/c mice) 1 h after injection with phosphate buffer saline. Compound names in red represent the predominant metabolites in the NMR spectra of each type of tissue. * Cut-off spectral region due to water suppression. 3-Letter codes are used for amino acids; Acet: acetate; Ado: adenosine; ADP: adenosine diphosphate; AMP: adenosine monophosphate; ATP: adenosine triphosphate; Cho: choline; Cre: creatine; Glc: glucose; GPC: glycerophosphocholine; GSH: glutathione (reduced); Hip: hippurate; HX: hypoxanthine; Ino: inosine; Lac: lactate; NAD^+^: nicotinamide adenine dinucleotide (reduced); NAM: niacinamide; PC: phosphocholine; Tau: taurine; TMAO: trimethylamine *N*-oxide; UDP-GlcA: uridine diphosphate-glucuronate; Urd: uridine.

Figure 1 shows the average ^1^H NMR spectra of polar extracts obtained from the kidney, liver, and breast tissue of healthy mice (controls), including semi-quantitative indication of predominant metabolites (compounds noted in red). Table S1 presents the overall list of assigned peaks (52 in total) and corresponding information regarding metabolite predominance in each type of tissue. In general, our results are consistent with previous reports of ^1^H NMR spectra of kidney [44,45,46] and liver extracts [46,47,48,49], adding new information regarding the polar metabolome of mice kidney (namely, changes in the levels of inosine monophosphate (IMP), uridine monophosphate (UMP), allantoin, threonine, 3-hydroxyisobutyrate (3-HIBA), trimethylamine *N*-oxide (TMAO), uridine diphosphate-glucuronate (UDP-GlcA)), liver (asparagine, glycerophosphocholine (GPC), hypoxanthine, hippurate, IMP, threonine, 3-HIBA, TMAO, and UDP-GlcA), and breast tissue, for which, to our knowledge, no previous ^1^H NMR metabolomics study has been reported. By visual inspection of the spectra in Figure 1, it becomes clear that alanine, lactate, and taurine predominate in all three tissue types, whereas specific characteristics are noted, as expected, for different tissues: (1) kidney comprises relatively high levels of betaine, *m*-inositol, and inosine, (2) liver shows high levels of acetone, glucose, glycogen, and glutathione (reduced form, GSH), and (3) breast tissue shows high levels of creatine, phosphocholine, and glycerol moieties arising from glycerolipids. The following section describes the changes in the complex metabolic profiles of each tissue type upon time-course exposure to cDDP.

### 2.2. Impact of Mice Exposure to Cisplatin

#### 2.1.1. Kidney Metabolic Profiling

**Figure 2:**
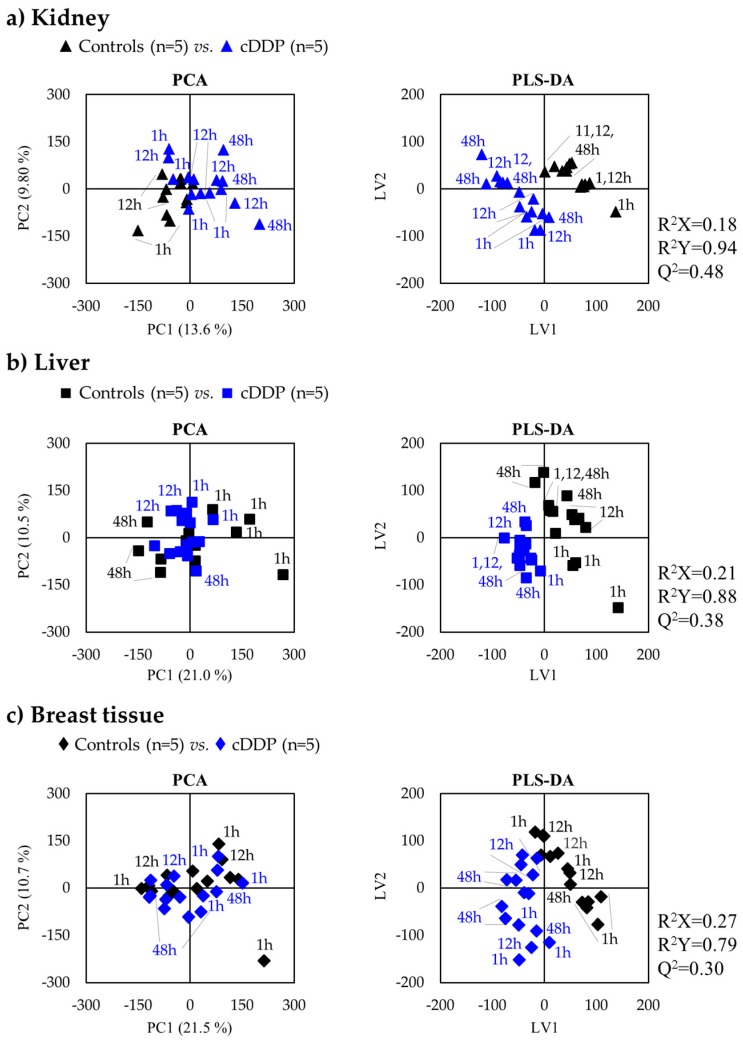
PCA (left) and PLS-DA (right) scores scatter plots for ^1^H NMR spectra of polar extracts of all time-course samples of (**a**) kidney, (**b**) liver, and (**c**) breast tissue of BALB/c mice.

Unsupervised analysis with PCA of the spectra of both controls and cDDP-exposed groups showed group separation only in the case of kidney (Figure 2, left), reflecting a stronger impact of the drug on the metabolic profile of that organ compared to liver and breast tissue. The corresponding PLS-DA score plots (Figure 2, right) confirmed such separation, given the satisfactory predictive power observed (Q^2^ = 0.48, compared to Q^2^ = 0.38 and 0.30 for liver and breast tissue, respectively). Particularly for kidney samples, it is interesting to note, through examination of PLS-DA plots, that both control and exposed kidney samples seemed to follow a slight time course tendency towards positive LV2, with relatively larger sample dispersion for the drug-exposed group.

**Figure 3:**
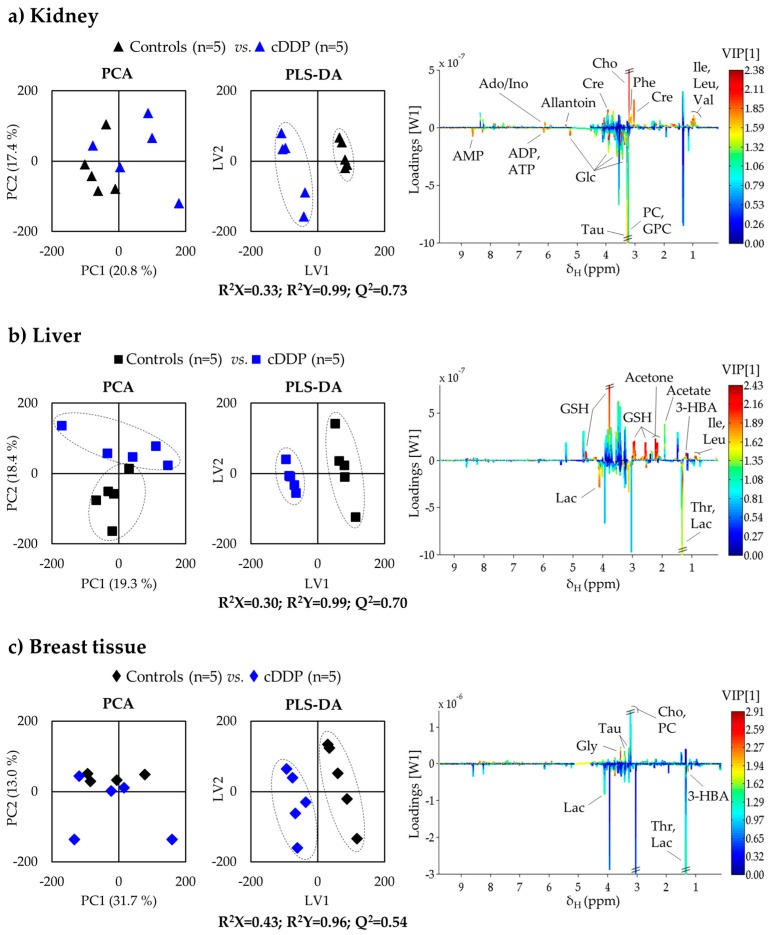
Scores scatter plots from PCA (left) and PLS-DA (middle) analysis obtained for ^1^H NMR spectra of (**a**) kidney, (**b**) liver, and (**c**) breast tissue of BALB/c mice at 12 h post-injection with cisplatin. LV1 loadings plots from PLS-DA analysis (right), colored according to variable importance to the projection (VIP) and with main peak assignment indicated. 3-Letter codes are used for amino acids; 3-HBA: 3-hydroxybutyrate; Ado: adenosine; ADP: adenosine monophosphate; AMP: adenosine monophosphate; ATP: adenosine monophosphate; Cho: choline; Cre: creatine; Glc: glucose; GPC: glycerophosphocholine; Ino: inosine; Lac: lactate; PC: phosphocholine; Tau: taurine.

**Figure 4:**
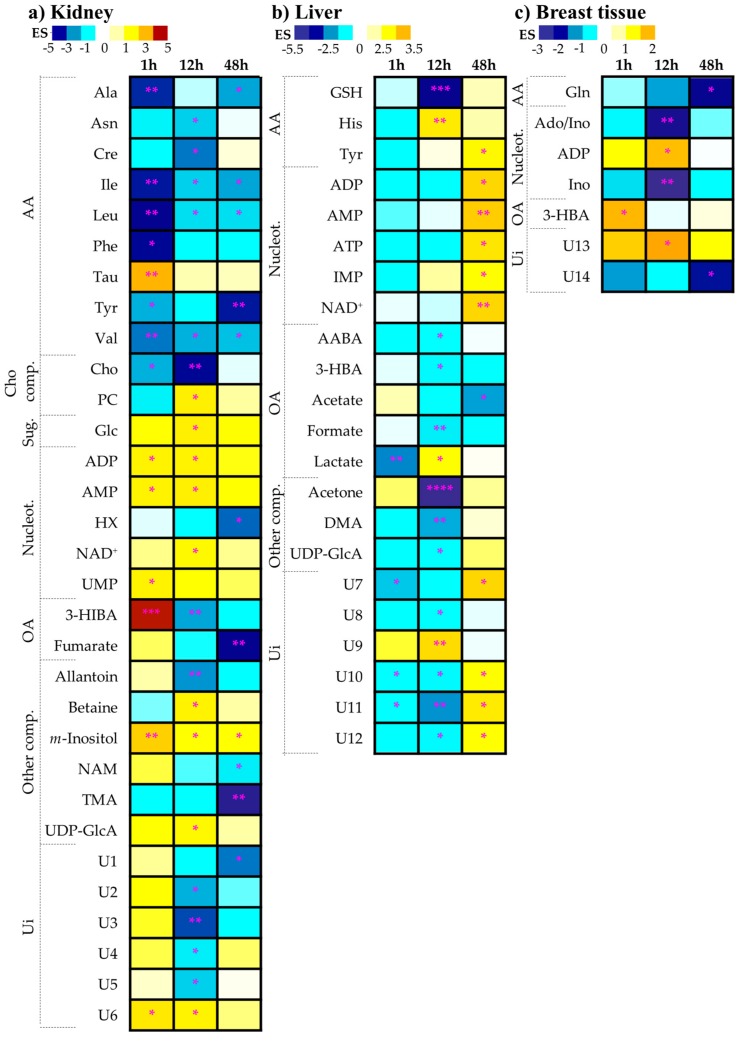
Heatmap illustrating the metabolic variations in (**a**) kidney, (**b**) liver, and (**c**) breast tissue of BALB/c mice at 1 h, 12 h, and 48 h post-injection with cisplatin, comparatively to the controls group. The scale is color-coded as a function of Effect Size, from minimum (dark blue) to maximum (dark red) values. Lines and columns represent metabolites and subjects, respectively. Asterisks represent the confidence interval of each variation: * *p*-value < 5.0 × 10^−2^; ** *p*-value < 1.0 × 10^−2^; *** *p*-value < 1.0 × 10^−3^; **** *p*-value < 1.0 × 10^−4^. 3-Letter codes are used for amino acids; ADP: adenosine diphosphate; AMP: adenosine monophosphate; Cho: choline; Glc: glucose; HX: hypoxanthine; NAD^+^: nicotinamide adenine dinucleotide (reduced); PC: phosphocholine; UMP: uridine monophosphate.

In order to systematically identify all the metabolites in kidney varying with exposure, at some stage of exposure, pairwise multivariate analysis was carried out for each timepoint (see results for t = 12 h in Figure 3). In spite of the low sample numbers, the PLS-DA model obtained for kidney tissue at 12 h post-injection showed high robustness (predictive power Q^2^ = 0.73), together with sample groups for 1 h (Q^2^ = 0.81, not shown), both showing better separation than for 48 h (Q^2^ = 0.54, not shown), which seems to indicate some degree of recovery from the shorter-term impact of cDDP. The underlying varying metabolites were identified through inspection of the LV1 loading plots corresponding to each time (e.g., for 12 h, as shown in Figure 3a, right), and their variation quantified by integration (Table 1). A set of 14 metabolites (including unassigned spin system U6) described the short-term impact of cDDP on kidney tissue, followed by an enlarged signature of 21 compounds at 12 h post-injection, and by a lesser number of changes (11-metabolite signature) at 48 h. The heatmap of these variations, organized by compound families (Figure 4), clearly shows that kidney is the most affected organ in terms of metabolism, compared to liver and breast tissue. Hence, its close examination will facilitate the identification of the short- and longer-term effects of cDDP (in the study range of 1–48 h), as well as metabolic recoveries and persistent deviations. Also, as this is, to our knowledge, the first NMR metabolomics study of cDDP exposure in mouse kidney, the present results provide additional information to existing MS-based reports on healthy animals [3,42,43], namely regarding cDDP-induced changes in 3-HIBA (↑↓), allantoin (↓, 12 h), adenosine monophosphate (AMP) (↑), betaine (↑,12 h), choline (↓), fumarate (↓, 48 h), *m-*inositol (↑), phosphocholine (PC) (↑, 12 h), taurine (↑, 1 h), trimethylamine (TMA) (↓, 48 h), and UDP-GlcA (↑, 12 h) (see Table 1).

The shorter-term (1 h) effects of cDDP on kidney comprise a general decrease in amino acids, with the exception of taurine (which increases significantly), in tandem with decreased free choline, and increased levels of ADP/AMP/UMP, 3-HIBA, and *m-*inositol (see Table 1, Figure 4a). Medium-term effects (12 h) involve a wider range of compounds, including additional decreases in asparagine, creatine, allantoin, 3-HIBA (inversed variation in relation to 1 h), and many unassigned resonances, as well as increased levels of PC, glucose, nicotinamide adenine dinucleotide (NAD^+^), betaine, and UDP-GlcA. The column corresponding to 48 h shows that many metabolites approach levels characteristic of controls, as illustrated by white or light colors without statistical relevance (Figure 4). More persistent amino acid variations relate to decreased alanine, tyrosine, and the three BCCAs. Other longer-term deviant levels comprise decreased levels of hypoxanthine, fumarate, niacinamide (NAM), TMA, unassigned resonance U1 (δ 0.89, triplet), and increased *m-*inositol.

#### 2.1.2. Liver Metabolic Profiling

In the case of liver, this is, to the best of our knowledge, the first metabolomics study of liver tissue exposed to cDDP using either NMR or MS. In liver, PCA and PLS-DA scores show either no group separation (PCA, see Figure 2b, left) or weak separation in supervised analysis (see Figure 2b, right). In PLS-DA, a slight time-course tendency is hinted at in controls, but no clear time tendency is seen in drug-exposed liver. Interestingly, this group shows the least dispersion in PLS-DA scores among all three treated tissues (see Figure 2, right), thus reflecting the higher tissue homogeneity of liver, and suggesting that liver exposure to cDDP seems to be the least time-dependent. At 12 h post-injection, a separation is clear in both PCA and PLS-DA scores (Q^2^ = 0.70) (Figure 3b), and the profile illustrated in the corresponding loadings plot (Figure 3b, right) reflects changes in some of the specific predominant metabolites in liver, namely lactate, GSH, and ketone bodies.

The current results show that the short-term effects of cisplatin in liver are limited to decreases in lactate and threonine (as previously reported for human liver cell line L02 exposed to cDDP [27]), to which we add three decreased unassigned resonances (U7 δ 0.73 singlet, U10, δ 4.31 doublet, and U11, δ 6.18 singlet), with U7 tentatively assigned to bile acids (Table 2). The stronger effects of cDDP on the liver metabolic profile take place at 12 h with a 15-metabolite signature, followed by recovered levels for most of those metabolites at 48 h and longer-term novel variations in 7 metabolites (in addition to reversed changes in unassigned resonances U10 to U12) (see Table 2). Inspection of the corresponding heatmap (Figure 4b) shows that visible metabolite variations tend to generally comprise decreases at 1 and 12 h (note GSH and acetone marked decreases at 12 h, in particular), with few exceptions of increasing tendencies (note increased histidine and lactate at 12 h). Conversely, at 48 h, there is a clear predominance of increased metabolite levels in all families, except organic acids (namely, note the significant acetate decrease at 48 h). Hence, the dynamics of cDDP exposure seems to be slightly delayed in liver compared to kidney, with most changes occurring at 12 h post-injection and beyond. Notably, the most marked persistent changes at 48 h comprise increased levels of tyrosine, ADP/AMP/ATP/IMP/NAD^+^, and four unassigned resonances, in tandem with an acetate decrease.

#### 2.1.3. Breast Tissue Metabolic Profiling

Breast tissue is shown to exhibit the least affected metabolic profile following treatment with cisplatin (Figure 2c), with no clear time course trend, which may also reflect the higher heterogeneity of this tissue. However, it is worthy of note that a slightly higher number of metabolite changes take place at 12 h post-injection, compared to 1 and 48 h (Table 3). The first indication of deviant metabolism is increased levels of the ketone body 3-hydroxibutyrate (3-HBA), which, however, quickly levels out back to control levels at 12 h, when decreased inosine and increased ADP levels are also noted (in tandem with an increase in U13, δ 1.25, singlet). All these changes are, however, restored to control values at 48 h, and only glutamine and U14 (δ 7.68, singlet) remain at lower levels, compared to controls. To our knowledge, this is the only study to date to have reported on drug-elicited, metabolic changes in breast tissue. As this type of tissue is mainly composed of lipids, the additional analysis of the lipophilic metabolome would certainly be of interest. However, the polar metabolome changes reported here already advance the early importance of 3-HBA, followed by a nucleotide deviant response, and finally, an important role played by glutamine in the later stages (see Figure 4c).

## 3. Discussion

Regarding the effect of cDDP on mouse organ metabolism, this work is, to the best of our knowledge, the first NMR-based metabolomics study. In the case of kidney metabolism, as a complementary strategy to MS-based metabolomics (in terms of higher holistic nature and reproducibility, simpler non-destructive sample handling, albeit lower sensitivity), this NMR work complements previous MS studies of mice kidney extracts carried out to characterize cDDP effects [3,42,43]. Furthermore, the present work considers effects as early as 1 h post-injection for all three tissues types, a time range, to the best of our knowledge, only comparable to that of one previous MS-based report on kidney exposed to cDDP ([3], 3–48 h).

In terms of the relative dynamics of the metabolic effects, it was observed that kidney suffers a large impact of the drug at only 1 h, evidencing the strongest short-term effect within the different tissues considered. At 1 h, the general amino acids decrease noted in kidney tissue is consistent with previous reports that employed cDDP doses of similar orders of magnitude (0.5 to 10 mg/mL, [42,43]), although tissue metabolic changes were measured within the timescale of days. One such study showed that a tandem increase in amino acids occurs in urine; this was interpreted as evidence of reabsorption impairment of amino acids in kidney due to nephrotoxicity and, thus, inducing increased amino acid excretion. However, the authors [43] also note some exceptions to the tissue decreasing levels tendency, e.g., for glutamine, similar to our observation regarding taurine. On the other hand, another recent report using a combination of MS imaging and LC-MS [3] indicated a general amino acid increase upon drug administration at times as short as 3 h, again with some exceptions (e.g., decreases in glutamate, glutamine, and cysteine). The latter study, however, involved the administration of a 10-fold higher cDDP concentration (30 mg/kg), which suggests that a strong dose-dependent effect on amino acid levels occurs, from decreased levels, at doses of a few mg/kg, to increased general levels, at much higher cDDP doses. Incidentally, U-shaped variations in metabolite levels have also been reported for human liver cell line, subjected to cDDP in the 1 nM–1 mM range [27], suggesting that similar behavior may be observed in vivo (namely in kidney). It is interesting to note, however, that in spite of the two previous, apparently contradicting studies in relation to amino acids levels in kidney tissue [3,43], glutamate levels behave similarly, in that they oppose the trend of most amino acids. In the present study, we noted taurine as differing from the decreased overall trend, which is consistent with a biochemical proximity between taurine and glutamate, as suggested before [3] for glutamate/hypotaurine/cysteine, all of which are involved in taurine and hypotaurine metabolism. In addition, cysteine (and methionine) metabolism is associated with glutathione metabolism, where GSH plays an important role in anti-oxidative mechanisms. Other early effects of cDDP on kidney comprise increased *m*-inositol, adenine, and uridine nucleotides, namely ADP/AMP/UMP, the latter in apparent contradiction to the recent high-dose cDDP report [3], again evidencing an apparent reversal effect induced by larger doses, as noted for amino acids. At 12 h post-injection, there is evidence of a deviant behavior in energy metabolism (glucose, creatine), cell membrane metabolism (choline and PC), and glycosylation metabolism (UDP-GlcA), in addition to a large number of still unassigned compounds. This strong deviant signature is mostly reversed to controls at 48 h, with the exception of a few compounds, including alanine, tyrosine, and the three BCCAs. Increased levels of the latter were particularly noted to be a strong excretory marker of cDDP renal toxicity [42], consistent with impaired reabsorption of amino acids and their subsequent decreased levels in the tissue, which we confirm for cDDP doses of a few mg/kg. Alanine decrease stands out as a possible global indicator of longer-term cDDP-induced nephrotoxicity, since it was also noted by MS metabolomics of kidney extracts in melanoma tumor-bearing animals treated with cDDP for 21 days [22]. Deviant levels of hypoxanthine, fumarate, *m*-inositol, NAM (vitamin B3), and TMA remain at 48 h as relatively late effects of cDDP. The latter two compounds may be indicative of an abnormal gut microflora metabolic response to cDDP, consistent with previous reports of dysbiotic intestinal microflora in relation to kidney disease [52].

The early amino acid depletion in kidney is accompanied by a qualitative (non-significant) tendency for amino acids decrease also in liver, although statistically relevant effects are few, and are related to decreased lactate levels and a few other unassigned resonances. The major impact of cDDP on liver takes place at 12 h and 48 h, but in a distinct way: at 12 h, ketone bodies (acetone and 3-HBA) seem to be used up for enhanced energy production; this may justify the enhanced levels of AMP/ATP/IMP/NAD^+^ noted at 48 h post-injection. The profile in unassigned resonances shows a general reversal from 12 to 48 h, which in itself is interesting as part of the characteristic signatures, thus supporting further endeavors to identify these resonances. In the case of liver, this is, to the best of our knowledge, the first NMR metabolomics study of liver tissue exposed to cDDP; the only comparison possible with previous results refers to the human liver L02 cell line [27]. In cells, however, cDDP seems to induce extensive changes in many amino acid levels, not observed here, while no changes are noted in ketone bodies. The same study does show some changes in lactate and GSH levels depending on cDDP doses, which suggests that lactate/GSH changes may be translational to in vivo.

Finally, in tandem with the early effects on kidney and liver discussed above, the sole early change in breast tissue (increased ketone body 3-HBA) may reflect a deviant shift to enhanced fatty acid oxidation which quickly leveled off subsequently. The strong nitrogenated bases decrease at 12 h, and the usage of glutamine at 48 h requires further investigation, although we propose at this point that nucleotide metabolism and glutamine usage, possibly to sustain energy production, may contribute to the metabolic breast tissue signature of cDDP response.

## 4. Materials and Methods

### 4.1. Chemicals

Cisplatin (cis-dichlorodiammine platinum (II), 99.9%) was purchased from Sigma-Aldrich (Sintra, Portugal). Euthasol^®^ solution (400 mg/mL pentobarbital sodium) was from Le Vet (Oudewater, Netherlands). All reagents were of analytical grade.

### 4.2. Ethical Considerations

Handling and care of animals were consistent with Portuguese (Decreto-Lei n.°113/2013) and European legislation (Directive 2010/63/EU) on the protection of animals used for scientific purposes, and were in agreement with the recommendations in the Guide for Care and Use of Laboratory Animals of the National Institutes of Health (NIH). The study protocol was approved by the Ethics Committee for Animal Experimentation of the Faculty of Pharmacy of the University of Porto, Porto, Portugal (Permit Number: 25-10-2015), and by the Ethics Committee and the Organ Responsible for the Welfare of Animals of ICBAS-UP, Porto, Portugal (Permit number 134/2015). The ARRIVE Guidelines were followed for reporting in vivo experiments [53].

### 4.3. Animals

Six-week old, Specific-Pathogen-Free (SPF), female BALB/cByJ mice (30 animals in total) were purchased from Charles River Laboratories (L’Arbresle, France) and acclimatized for 1 week at ICBAS-UP Rodent Animal House Facility (Porto, Portugal). Animals were randomly distributed in groups of five per cage (in individually ventilated cages) with enrichment material (corncob bedding, paper role tube, and one large sheet of tissue paper for nesting), and were housed in a SPF environment with *ad libitum* access to water and standard pellet food under controlled 12 h light/dark cycles (lights on at 7.00 AM), temperature (22 ± 2 °C), and humidity (50 ± 10%).

### 4.4. In Vivo Experimental Procedures

After 1 week of acclimatization, animals were randomly allocated into two groups (15 animals per group): cDDP and vehicle (PBS). At the beginning and end of the experiments, the animals weighed 19.9 ± 1.5 g and 20.0 ± 1.6 g, respectively. CDDP 3.5 mg/kg solution in PBS: H_2_PO_4_ 1.5 mM, Na_2_HPO_4_ 4.3 mM, KCl 2.7 mM, NaCl 150 mM, pH 7.4 and PBS alone were administered in a single dose, via intraperitoneal injection (volume of injection = 200 µL) in the cDDP and vehicle groups, respectively. After drug/vehicle administration, five animals from each group were euthanized, at each time-point (1, 12, and 48 h), with pentobarbital intraperitoneal injection (120 mg/kg) followed by cardiac puncture. The left kidney, median liver lobe, and thoracic mammary glands were excised and snap frozen in liquid nitrogen and stored at −80 °C for NMR analysis. All injected solutions were sterile filtered. No biochemical measurements were performed to detect kidney damage, as the volume of blood obtained was too small (under 400 µL and no sample, in some cases). In any case, all animals showed no loss of weight (see the weight values above), no evidence of decreased food or drink intake, and no behavioral changes whatsoever, which indicated the absence of significant kidney damage. This was also supported by identical observations in a small pilot study (*n* = 2) of our own with animals injected with 3 cycles of cDDP (3.5 mg/kg per cycle), and by a previous report that blood urea nitrogen (BUN) in mice increased after 24 h for a 30 mg/kg b.w. cDDP dose [3].

### 4.5. Sample Preparation for NMR

Tissues were weighted (*ca.* 50, 60, and 35 mg for kidney (superior half of organ), liver (median liver lobe), and breast tissue (predominantly consisting of mammary gland), respectively) and ground by mechanical maceration with cooled liquid N_2_ using a pestle and mortar [54,55,56]. Tissue extracts were then prepared using the biphasic methanol/chloroform/water (2.0:2.0:1.0) procedure [57]. Briefly, samples were homogenized in 8.0 mL/g cold 80% methanol, 4.0 mL/g cold chloroform, and 2 mg/L cold water, vortexed for 60 s and kept on ice for 10 min [57]. Samples were centrifuged (8000 rpm, 5 min, 23 °C), and polar phases were removed, vacuum-dried, and stored at −80 °C until analysis. Before NMR acquisition, aqueous extracts were suspended in 650 µL of 100 mM sodium phosphate buffer (pH 7.4, in D_2_O containing 0.25% 3-(trimethylsilyl)-propionic-2,2,3,3-d_4_ acid (TSP, for chemical shift referencing)), homogenized, and 600 µL of the resulting solution was then transferred to NMR tubes.

### 4.6. NMR Measurements

All NMR spectra were acquired on a Bruker AVANCE III spectrometer operating at 500.13 MHz for ^1^H. The unidimensional (1D) proton NMR spectra of the aqueous extracts were recorded at 298 K, using the “noesypr1d” pulse sequences (Bruker library), with 2.34 s acquisition time, 2 s relaxation delay, 512 scans, 7002.801 Hz spectral width, and 32 k data points. Each free-induction decay was zero-filled to 64 k points and multiplied by a 0.3 Hz eponential function prior to Fourier transformation. Spectra were manually phased, baseline-corrected, and chemical-shift referenced to TSP. Bidimensional (2D) NMR homonuclear (TOCSY) and heteronuclear (HSQC) spectra were recorded for selected samples to aid spectral assignment. Peak assignment was based on comparison with data available on Bruker BBIOREFCODE spectral database and the human metabolome database (HMDB) [58], as well as on existing literature.

### 4.7. Data Processing and STATISTICS

The 1D NMR spectra were converted into data matrices (AMIX-viewer 3.9.14, BrukerBiospin, Rheinstetten, Germany) prior multivariate analysis, and exclusion of the water (δ 4.5–5.2) and methanol (singlet at δ 3.36) regions was applied. Spectra were aligned by recursive, segment-wise peak alignment (RSPA) (Matlab 8.3.0, The MathWorks Inc., Natick, Massachusetts, USA) and normalized to spectral total area, to account for sample concentration differences. Multivariate analysis of the 1D spectra was carried out using principal component analysis (PCA) and partial least-squares discriminant analysis (PLS-DA) after unit variance (UV) scaling of the spectra (SIMCA-P 11.5; Umetrics, Umeå, Sweden). PLS-DA loadings were back-transformed, multiplying each variable by its standard deviation, and colored according to variable importance to the projection (VIP) (Matlab 8.3.0, The MathWorks Inc., Natick, Massachusetts, USA). PLS-DA models were considered statistically robust for predictive power (Q^2^) values ≥ 0.05. The relevant resonances identified in PLS-DA loadings plots were integrated (Amix 3.9.5, Bruker BioSpin, Rheinstetten, Germany), normalized, and variations assessed by univariate analysis (Shapiro-Wilk test used to assess if data was normally distributed, Student’s *t* test used for normally-distributed data, or Wilcoxon test used for non-normally distributed data) (*R*-statistical software). Significantly changed metabolites (*p* < 0.05) were identified, and the corresponding effect size values calculated [50]. False discovery rate (FDR) correction based on the Benjamini and Hochberg method [51] was used to correct *p*-values for multiple comparisons.

## 5. Conclusions

This work describes, to the authors´ knowledge, the first NMR metabolomics analysis of mice kidney, liver, and breast tissue in response to cisplatin exposure (single 3.5 mg/kg injection), to search for early metabolic signatures of cDDP biotoxicity. The results obtained show that the largest metabolic impact occurs for kidney, as expected, as early as at 1 h post-injection. Kidney shows a marked depletion in amino acids, comprising a 14-metabolite signature at 1 h. The highest number of variations is noted at 12 h after drug administration, although many of these recover to control levels at 48 h, with the exception of persistent changes in 10 specific metabolites (namely, alanine, BCCAs, tyrosine, hypoxanthine, fumarate *m-*inositol, niacinamide, and trimethylamine). Liver and breast tissue also show more marked metabolic changes at 12 h, with liver standing out for its persistent increase in the levels of several nucleotides as a longer-term cisplatin effect. The present study, however, presents some limitations, namely regarding some still unassigned resonances included in all tissue signatures (an observation transversal to most metabolomic studies, either NMR- or MS-based), and the lack of biochemical measurements of biotoxicity in both kidney and liver tissues (although there was no behavioral evidence of significant organ damage). In spite of these, this work successfully reports the very early (1–12 h) cisplatin effects in kidney, liver, and breast tissue metabolism, thus contributing to our understanding of multi-organ cDDP biotoxicity.

## Figures and Tables

**Table 1:** List of metabolite variations in kidney of mice exposed to cDDP at 1, 12, and 48 h post-injection, relative to the controls. Only variations with 95% significance level (*p*-value < 0.05) are shown; ES, effect size and corresponding error [50].

Metabolite	δ_H_ ppm (multiplicity)	1 h				12 h				48 h			
		ES	±	Error	*p*-Value	ES	±	Error	*p*-Value	ES	±	Error	*p*-Value
3-HIBA	1.09 (d)	4.7	±	2.5	1.9 × 10^−4 a^	−2.2	±	1.6	9.5 × 10^−3^	-	-	-	-
ADP ^c^	8.28 (s)	1.8	±	1.6	2.9 × 10^−2^	1.8	±	1.5	1.6 × 10^−2^	-	-	-	-
Ala ^b^	1.48 (d)	−3.0	±	1.9	9.5 × 10^−3 a^	-	-	-	-	−2.2	±	1.7	1.1 × 10^−2^
Allantoin	5.39 (s)	-	-	-	-	−2.3	±	1.6	9.3 × 10^−3^	-	-	-	-
AMP	8.61 (s)	1.8	±	1.5	3.3 × 10^−2^	1.9	±	1.5	2.8 × 10^−2^	-	-	-	-
Asn ^b, c^	2.96 (m)	-	-	-	-	−1.9	±	1.5	2.3 × 10^−2^	-	-	-	-
Betaine	3.90 (s)	-	-	-	-	1.8	±	1.5	3.8 × 10^−2^	-	-	-	-
Cho	3.21 (s)	−2.1	±	1.6	1.6 × 10^−2^	−3.2	±	1.9	2.7 × 10^−3^	-	-	-	-
Cre ^c^	3.93 (s)	-	-	-	-	−2.5	±	1.6	1.1 × 10^−2^	-	-	-	-
Fumarate	6.52 (s)	-	-	-	-	-	-	-	-	−3.5	±	2.1	2.7 × 10^−3 a^
Glc ^b^	5.23 (d)	-	-	-	-	1.9	±	1.5	2.7 × 10^−2^	-	-	-	-
Hypoxanthine ^b^	8.21 (s)	-	-	-	-	-	-	-	-	−2.6	±	1.8	3.3 × 10^−2^
Ile ^b, c^	1.01 (d)	−3.1	±	1.9	4.1 × 10^−3 a^	−1.9	±	1.5	3.3 × 10^−2^	−2.2	±	1.7	1.5 × 10^−2^
Leu ^b, c^	0.96 (t)	−3.5	±	2.1	1.2 × 10^−3 a^	−1.8	±	1.5	3.0 × 10^−2^	−1.8	±	1.6	2.5 × 10^−2^
*m*-Inositol	3.62 (t)	2.5	±	1.7	6.8 × 10^−3 a^	1.5	±	1.4	4.4 × 10^−2^	1.6	±	1.5	3.2 × 10^−2^
Niacinamide ^b^	7.60 (dd)	-	-	-	-	-	-	-	-	−1.7	±	1.5	3.6 × 10^−2^
PC	3.22 (s)	-	-	-	-	1.9	±	1.5	1.7 × 10^−2^	-	-	-	-
Phe ^b,c^	7.33 (d)	−3.2	±	2.0	1.6 × 10^−2^	-	-	-	-	-	-	-	-
Tau	3.42 (t)	3.0	±	1.9	3.2 × 10^−3 a^	-	-	-	-	-	-	-	-
TMA	2.89 (s)	-	-	-	-	-	-	-	-	-4.3	±	2.4	1.1 × 10^−3 a^
Tyr ^b, c^	6.90 (d)	−2.1	±	1.6	1.6 × 10^−2^	-	-	-	-	-3.1	±	1.9	2.5 × 10^−3 a^
NAD^+ c^	8.43 (s)	-	-	-	-	1.7	±	1.4	1.6 × 10^−2^	-	-	-	-
UDP-GlcA	7.95 (d)	-	-	-	-	1.7	±	1.4	3.0 × 10^−2^	-	-	-	-
UMP ^c^	5.99 (m)	1.8	±	1.6	2.8 × 10^−2^	-	-	-	-	-	-	-	-
Val ^b, c^	1.05 (d)	−2.5	±	1.7	8.9 × 10^−3 a^	−2.1	±	1.5	2.0 × 10^−2^	−2.0	±	1.6	2.1 × 10^−2^
U1	0.89 (t)	-	-	-	-	-	-	-	-	−2.5	±	1.8	1.6 × 10^−2^
U2	0.93 (s)	-	-	-	-	−2.1	±	1.6	1.2 × 10^−2^	-	-	-	-
U3	1.62 (d)	-	-	-	-	−2.8	±	1.7	5.0 × 10^−3^	-	-	-	-
U4	2.92 (s)	-	-	-	-	−1.7	±	1.4	3.5 × 10^−2^	-	-	-	-
U5	3.15 (s)	-	-	-	-	−1.9	±	1.5	3.2 × 10^−2^	-	-	-	-
U6	3.35 (s)	2.0	±	1.6	1.8 × 10^−2^	1.8	±	1.5	2.2 × 10^−2^	-	-	-	-

^a^ Metabolic variations remain significant after False Discovery Rate correction [51]; ^b^ metabolite variations reported previously [3,42,43], ^c^ metabolite variations noted in different direction compared to previous report [3]. 3-Letter codes are used for amino acids; 3-HIBA: 3-hydroxyisobutyrate; ADP: adenosine diphosphate; AMP: adenosine monophosphate; Cho: choline; Cre: creatine; Glc: glucose: PC: phosphocholine; Tau: taurine; TMA: trimethylamine; NAD^+^: nicotinamide adenine dinucleotide; UDP-GlcA: uridine-diphosphate-glucoronate; Ui: unassigned i; UMP: uridine monophosphate.

**Table 2:** List of metabolite variations in liver of mice exposed to cDDP at 1, 12 and 48 h post-injection, relatively to the controls. Only variations with 95% significance level (*p*-value < 0.05) are shown; ES, effect size and corresponding error [50].

Metabolite	δ_H_ ppm (multiplicity)	1 h				12 h				48 h			
		ES	±	Error	*p*-Value	ES	±	Error	*p*-Value	ES	±	Error	*p*-Value
2-aminobutyrate ^†^	0.80 (t)	-	-	-	-	−1.6	±	1.4	4.4 × 10^−2^	-	-	-	-
3-HBA	1.20 (d)	-	-	-	-	−1.8	±	1.5	3.1 × 10^−2^	-	-	-	-
Acetate ^b^	1.92 (s)	-	-	-	-	-	-	-	-	−2.5	±	1.7	2.0 × 10^−2^
Acetone	2.24 (s)	-	-	-	-	−5.1	±	2.6	5.2 × 10^−5a^	-	-	-	-
ADP	8.54 (s)	-	-	-	-	-	-	-	-	2.6	±	1.8	1.6 × 10^−2^
AMP ^b^	4.51 (dd)	-	-	-	-	-	-	-	-	2.8	±	1.8	3.8 × 10^−3^
ATP ^b^	8.52 (s)	-	-	-	-	-	-	-	-	2.3	±	1.7	1.6 × 10^−2^
DMA	2.73 (s)	-	-	-	-	−2.4	±	1.6	6.0 × 10^−3 a^	-	-	-	-
Formate	8.46 (s)	-	-	-	-	−1.9	±	1.5	7.9 × 10^−3 a^	-	-	-	-
GSH	2.55 (m)	-	-	-	-	−4.0	±	2.1	2.7 × 10^−4 a^	-	-	-	-
His	7.08 (s)	-	-	-	-	2.2	±	1.6	9.110^−3^	-	-	-	-
IMP	8.58 (s)	-	-	-	-	-	-	-	-	1.7	±	1.5	3.2 × 10^−2^
Lactate ^b^	4.10 (q)	−2.7	±	1.7	6.9 × 10^−3^	1.7	±	1.5	3.0 × 10^−2^	-	-	-	-
NAD^+^	8.43 (s)	-	-	-	-	-	-	-	-	2.6	±	1.8	9.9 × 10^−3^
Tyr	6.90 (d)	-	-	-	-	-	-	-	-	1.9	±	1.6	2.3 × 10^−2^
UDP-GlcA	7.95 (d)	-	-	-	-	-1.7	±	1.4	3.0 × 10^−2^	-	-	-	-
U7	0.73 (s)	−2.2	±	1.6	1.6 × 10^−2^	-	-	-	-	2.6	±	1.8	1.6 × 10^−2^
U8	0.85 (t)	-	-	-	-	−1.6	±	1.4	1.6 × 10^−2^	-	-	-	-
U9	3.10 (d)	-	-	-	-	2.5	±	1.7	9.8 × 10^−3 a^	-	-	-	-
U10	4.31 (d)	−1.6	±	1.4	4.7 × 10^−2^	−1.8	±	1.5	2.1 × 10^−2^	1.7	±	1.5	3.3 × 10^−2^
U11	6.18 (s)	−1.7	±	1.5	3.6 × 10^−2^	−2.6	±	1.7	7.8 × 10^−3 a^	2.2	±	1.7	2.3 × 10^−2^
U12	8.28 (br)	-	-	-	-	−1.8	±	1.5	3.5 × 10^−2^	1.8	±	1.5	4.2 × 10^−2^

^a^ Metabolic variations remaining significant after False Discovery Rate correction [51]; ^b^ metabolite variations observed in common with a previous study on the human liver cell line L02 [27] as, to our knowledge, no previous reports have been found in relation to liver tissue, either of murine or human nature; ^†^ Tentative of assignment. 3-HBA: 3-hydroxybutyrate; ATP: adenosine triphosphate; DMA: dimethylamine; GSH: glutathione (reduced) IMP: inosine monophosphate; other abbreviations are as defined in Table 1.

**Table 3:** List of metabolite variations in breast tissue of mice exposed to cDDP at 1, 12, and 48 h post-injection, relatively to controls. Only variations with 95% significance level (*p*-value < 0.05) are shown; ES, effect size and corresponding error [50]; ª Metabolic variations remaining significant after False Discovery Rate correction [51]. Ado: adenosine; Ino: inosine; other abbreviations are as defined in the legends of Table 1 and Table 2.

Metabolite	δ_H_ ppm (multiplicity)	1 h				12 h				48 h			
		ES	±	Error	*p*-value	ES	±	Error	*p*-value	ES	±	Error	*p*-value
3-HBA	1.20 (d)	1.8	±	1.5	4.3 × 10^−2^	-	-	-	-	-	-	-	-
Ado/Ino	6.10 (d)	-	-	-	-	−2.4	±	1.6	6.1 × 10^−3a^	-	-	-	-
ADP	8.54 (s)	-	-	-	-	1.7	±	1.4	3.1 × 10^−2 a^	-	-	-	-
Gln	2.45 (m)	-	-	-	-	-	-	-	-	−2.2	±	1.6	1.2 × 10^−2^
Ino	8.35 (s)	-	-	-	-	−2.8	±	1.7	2.4 × 10^−3 a^	-	-	-	-
U13	1.25 (s)	-	-	-	-	2.0	±	1.5	2.6 × 10^−2 a^	-	-	-	-
U14	7.68 (s)	-	-	-	-	-	-	-	-	−1.9	±	1.6	3.1 × 10^−2^

## Supplementary Materials

The following is available online at https://www.mdpi.com/2218-1989/9/11/279/s1, Table S1: List of compounds and corresponding spin systems identified in the 500 MHz ^1^H NMR spectra of aqueous extracts of kidney, liver and breast tissue of healthy BALB/c mice.
