# Supplementary material for: Multi-Organ NMR Metabolomics to Assess In Vivo Overall Metabolic Impact of Cisplatin in Mice
Source: Metabolites. 2019 Nov 13;9(11):279. doi: 10.3390/metabo9110279 (PMC6918135; doi:10.3390/metabo9110279)
Supplement: Supplementary file 1 [file metabolites-09-00279-s001.pdf]

Table S1. List of compounds and corresponding spin systems identified in the 500MHz ^{1}H NMR spectra of aqueous extracts of kidney, liver and breast tissue of BALB/c mice (control group). Symbols represent the detection of each metabolite on tissues' spectra: ✓, detectable; ✓✓ predominant; × absent. [a] Possible contaminants resulted from the extraction procedure. [†] Tentative of assignment. Abbreviations: s, singlet; d, doublet; t, triplet; q, quartet; m, multiplet; dd, double doublet; 3-HBA, 3-hydroxybutyrate; 3-HIBA, 3-hydroxyisobutyrate; ADP, adenosine diphosphate; AMP, adenosine monophosphate; ATP, adenosine triphosphate; DMA, dimethylamine; GPC, glycerophosphocholine; GSH, glutathione (reduced); IMP, inosine monophosphate; NAD+, nicotinamide adenine dinucleotide (reduced); PC, phosphocholine; UDP-GlcA, uridine diphosphate - glucuronate; UMP, uridine monophosphate; TMA, trimethylamine; TMAO, trimethylamine *N*-oxide.

Metabolite assignment	δ_H ppm (multiplicity, assignment)	HMDB ID [58]	K	L	BT
**Amino acids and derived compounds**					
Alanine	1.48 (d, βCH_3); 3.78 (q, αCH)	HMDB0000161	✓✓	✓✓	✓✓
Asparagine	2.84 (m, βCH_2); 2.96 (m, $\beta' CH_2$); 4.00 (dd, αCH)	HMDB0000168	✓	✓	✓
Aspartate	2.67 (dd, βCH); 2.80 (dd, β'CH); 3.90 (dd, αCH)	HMDB0000191	✓	✓	Res.
Creatine	3.04 (s, N-CH_3); 3.93 (s, N-CH_2)	HMDB0000064	✓	✓	✓✓
Glutamate	2.04 (m, βCH); 2.11 (m, β'CH); 2.35 (m, γCH_2); 3.74 (dd, αCH)	HMDB0000148	✓✓	✓	✓✓
Glutamine	2.13 (m, βCH_2); 2.45 (m, γCH_2); 3.77 (t, αCH)	HMDB0000641	✓	✓✓	✓✓
Glycine	3.56 (s, αCH_2)	HMDB0000123	✓✓	✓	✓✓
GSH	2.16 (m, βCH_2 Glu); 2.55 (m, γCH_2 Glu); 2.96 (m, αCH_2 Cys); 3.78 (αCH Glu); 4.16 (γCH_2 Cys); 4.57 (m, βCH_2 Cys); 8.37 (NH Gly); 8.56 (NH Cys)	HMDB0000125	×	✓✓	✓
Histidine	3.10 (dd, βCH2); 3.91 (dd, αCH); 7.08 (s, C4H ring); 7.90 (s, C2H ring)	HMDB0000177	✓	✓	✓
Isoleucine	0.94 (t, δCH3); 1.01 (d, β'CH3); 1.47 (m, γ'CH2); 1.99 (m, βCH); 3.67 (d, αCH)	HMDB0000177	✓	✓	✓
Leucine	0.96 (t, δCH_3/ $\delta' CH_3$); 1.71 (m, βCH_2/ γCH); 3.73 (t, αCH)	HMDB0000687	✓	✓	✓
Lysine	1.45 (m, γCH_2); 1.70 (m, δCH_2); 1.89 (m, βCH_2); 3.01 (t, εCH_2); 3.74 (t, αCH)	HMDB0000182	✓	✓	✓
Phenylalanine	3.99 (dd, αCH); 7.33 (d, C2H/ C6H ring); 7.38 (m, C4H ring); 7.42 (t, C3H/ C5H ring)	HMDB0000159	✓	✓	✓
Taurine	3.26 (t, S-CH_2); 3.42 (t, N-CH_2)	HMDB0000251	✓✓	✓✓	✓✓
Threonine	1.33 (d, γCH_3); 3.58 (d, αCH); 4.24 (m, βCH)	HMDB0000167	✓	✓	✓
Tyrosine	3.06 (m, $\beta' CH_2$); 3.95 (m, αCH); 6.90 (d, C3H/ C5H ring); 7.20 (d, C2H/ C6H ring)	HMDB0000158	✓	✓	✓
Valine	0.99 (d, γCH_3); 1.05 (d, $\gamma' CH_3$); 2.27 (m, βCH); 3.61 (d, αCH)	HMDB0000883	✓	✓	✓
**Choline compounds**					
Choline	3.21 (s, $N(CH_3)_3$); 4.07 (m, $\underline{CH_2}(OH)$)	HMDB0000097	✓✓	✓	✓✓
GPC	3.23 (s, $N(CH_3)_3$); 3.94 (m, αCH_2); 4.33 (m, PO_3-αCH_2)	HMDB0000086	✓✓	✓	✓✓

PC	3.22 (s, $N(CH_3)_3$); 4.17 (m, PO_3-CH_2)	HMDB0001565	✓	✓	✓✓
**Sugars**					
α-Glucose	3.41 (t, C5H); 3.53 (dd, C2H); 3.71 (t, C3H); 3.76 (dd, C6H); 3.83 (m, C4H); 3.83 (m, C6H'); 5.23 (d, C1H)	HMDB0003345	✓	✓✓	✓
β-Glucose	3.23 (dd, C2H); 3.40 (t, C5H); 3.47 (dd, C4H); 3.49 (t, C3H); 3.71 (dd, C6H'); 3.89 (m, C6H); 4.65 (d, C1H)	HMDB0003345	✓	✓✓	✓
**Nucleotides and derived compounds**					
Adenosine	4.29 (q, C4'H ribose); 4.44 (dd, C3'H ribose); 6.10 (d, C1'H ribose); 8.12 (s, C8H ring); 8.27 (s, C2H ring)	HMDB0000050	✓	✓	✓
ADP	4.22 (m, C5'H_2 ribose); 4.38 (m, C4'H ribose); 6.15 (d, C1'H ribose); 8.28 (s, C2H ring); 8.54 (s, C8H ring)	HMDB0001341	✓	✓	✓
AMP	4.36 (dd, C4'H ribose); 4.51 (dd, C2'H ribose); 6.14 (d, C1'H ribose); 8.27 (s, C2H ring); 8.61 (s, C8H ring)	HMDB0000045	✓	✓	✓
ATP	4.22 (m, C5'H_2 ribose); 4.40 (m, C4'H ribose); 6.14 (d, C1'H ribose); 8.24 (s, C2H ring); 8.52 (s C8H ring)	HMDB0000538	✓	✓	✓
Hypoxanthine	8.19 (s, C2H); 8.21 (s, C8H)	HMDB0000157	✓	✓	✓
IMP	4.02 (m, C5'H_2); 4.51 (m, C3'H); 6.14 (d, C1'H); 8.58 (s, C8H ring)	HMDB0000175			
Inosine	3.90 (dd, C5'H_2); 4.26 (dd, C4'H); 4.44 (dd, C3'H ribose); 6.10 (d, C1'H); 8.18 (s, C8H ring); 8.35 (s, C2H ring)	HMDB0000195	✓✓	✓	✓
NAD^+	4.23 (m, A5'); 4.36 (m, A4'); 4.50 (m, A3'); 4.54 (m, N2'); 6.04 (d, N1'); 8.18 (s, A2); 8.19 (N5); 8.43 (s, A8); 8.83 (d, N4); 9.15 (d, N6); 9.34 (s, N2)	HMDB0000902	Res.	Res.	Res.
UMP	4.01 (m, C5'H_2 ribose); 4.37 (t, C3'H ribose); 4.43 (t, C2'H ribose); 5.99 (m, C6H ring)	HMDB0000288	Res.	✓	Res.
Uridine	4.23 (t, C3'H ribose); 4.38 (t, C2'H ribose); 5.90 (d, C5H ring); 5.94 (d, C1'H ring); 7.86 (d, C6H ring)	HMDB0000285	✓	✓	×
**Organic acids**					
2-aminobutyrate[†]	0.80 (t, CH_3)	HMDB0000452	✓	✓	✓
3-HBA	1.20 (d, CH_3); 2.31 (m, CH_2); 4.16 (m, CH)	HMDB0000357	✓	✓	✓
3-HIBA	1.09 (d, CH_3); 2.65 (m, CH)		✓	Res.	×
Acetate	1.92 (s, βCH_3)	HMDB0000042	✓	✓✓	✓✓
Formate	8.46 (s, CH)	HMDB0000142	✓	✓	✓
Fumarate	6.52 (s, CH)	HMDB0000134	Res.	✓	×
Hippurate	7.54 (m, C3H/ C5H ring); 7.62 (m, C4H ring); 7.82 (dd, C2H/ C6H ring)	HMDB0000714	✓	✓	×
Lactate	1.33 (d, CH_3); 4.10 (q, CH)	HMDB0000190	✓✓	✓✓	✓✓
Succinate	2.41 (s, CH_2)	HMDB0000254	✓✓	✓✓	✓
**Other compounds**					
Acetone	2.24 (s, CH_3)	HMDB0001659	✓	✓	✓
Allantoin	5.39 (s, CH)	HMDB0000462	Res.	Res.	×
Betaine	3.25 (s, CH_2); 3.90 (s $N(CH_3)_3$)	HMDB0000043	✓✓	✓	✓
DMA	2.73 (s, $(CH_3)_2$)	HMDB0000087	Res.	Res.	×
Glycerol moieties (glycerolipids)	3.55 (m, C1H_2); 3.64 (m, C2H_2); 3.77 (m, C3H_2)	HMDB0000131	×	×	✓✓

Glycogen	5.40 (br, CH)	HMDB0000757	✗	✓	✗
Ethanol[a]	1.19 (t, CH_3); 3.65 (q, CH_2)	HMDB0000108	✓	✓	✓
m-Inositol	3.28 (t, C5H); 3.62 (t, C4H/ C6H); 4.06 (t, C2H)	HMDB0000211	✓✓	✗	✓
Propylene glycol[a]	1.15 (d, CH_3); 3.87 (m, CH)	HMDB0001881	✓✓	✓✓	✓✓
Niacinamide	7.60 (dd, C5H ring); 8.25 (dd, C4H ring); 8.72 (dd, C6H ring); 8.94 (s, C2H ring)	HMDB0001406	✓	✓	✓
TMA	2.89 (s, $(CH_3)_3$)	HMDB0000906	✓	✓	Res.
TMAO	3.27 (s, CH_3)	HMDB0000925	✓	✓	✓
UDP-GlcA	5.61 (dd, C1H Glc); 7.95 (d, C2H Uridine)	HMDB0000935	Res.	✓	✗
